
JOURNAL INFORMATION
==============================
NLM Title Abbreviation: ScientificWorldJournal
Journal ID: ScientificWorldJournal
NLM Journal ID: 101131163
Journal ID: TSWJ

The Scientific World Journal

ISSN: 2356-6140
EISSN: 1537-744X
Publisher: Wiley

ARTICLE INFORMATION
==============================
PMCID: PMC6084208
PMID: 30147607
DOI: 10.1100/tsw.2001.227
Article ID: 712610
Article version: 1
Subjects: Short Report

Olomoucine II, New Effective CDK Inhibitor with Strong Cytotoxic Properties

Lenobel R. *
Havli L.
Kryscaron P.V.
Otyepka M.
Strnad M.
Laboratory of Growth Regulators, Institute of Experimental Botany ASCR & Palacký University, Šlechtitelů 11, 783 71 Olomouc, Czech Republic
*R. Lenobel: lenobel@aix.upol.cz
Publication date: 2001
Electronic publication date: 2001 Oct 17
Volume: 1
First page: 128
Last page: 128
Copyright: Copyright © 2001 R. Lenobel et al.
Copyright year: 2001
License: This is an open access article distributed under the Creative Commons Attribution License, which permits unrestricted use, distribution, and reproduction in any medium, provided the original work is properly cited.
License URL: https://creativecommons.org/licenses/by/3.0/

Funding: Ministry of Education, Youth and Sports MSM 153100008

==============================
Miami *Nature Biotechnology* Short Reports
*TheScientificWorld* (2001) 1 (S3), 128SR
ISSN 1532-2246; DOI 10.1100/tsw.2001.227

### OLOMOUCINE II, NEW EFFECTIVE CDK INHIBITOR WITH STRONG CYTOTOXIC PROPERTIES

R. Lenobel*, L. Havlíček, P.V. Kryštof, M. Otyepka, and M. Strnad

Laboratory of Growth Regulators, Institute of Experimental Botany ASCR & Palacký University, Šlechtitelů 11, 783 71 Olomouc; Czech Republic
* lenobel@aix.upol.cz

**INTRODUCTION.** The cell division cycle is controlled by cyclin-dependent kinases (cdk), which consist of a catalytic subunit (cdk1-cdk8) and a regulatory subunit (cyclin A - cyclin H). These proteins are regulated in several ways: subunit production, complex formation, (de)phosphorylation, cellular localization and interaction with various natural protein inhibitors. Recently, a deregulation of cdks has been proved in human primary tumors and in tumor cell lines. The discovery evoked a strong interest in inhibitors of cdk that could play a role in the therapy of cancers. Four types of cdk inhibitors have so far been described: staurosporine, flavopiridole (L86-8275), butyrolactone-1, and purine derivatives.

**METHODS.** Chemical synthesis was based on alkylation of 2,6-dichloropurine. Newly developed compounds were tested for cdk2/cyclin E inhibitory activity to determine the basic relationships between their interaction energy of docked complex and the inhibitory activity (1). Antiproliferative, cytotoxic and pro-differentiating capacity of olomoucine and its structural derivatives on the range of human and animal cell lines. We have been using the following cell lines: *HELA* (human cervical carcinoma), *MCF7* (human breast adenocarcinoma), *NIH 3T3* (mouse fibroblasts), *HOS* (human osteogenic sarcoma), *HL 60* (human promyelocytic leukemia), *G 361* (human malignant melanoma), *K562* (human erythroleukaemia), *CEM* (human lymphoblastoid leukaemia). Tested drugs were added to the cell cultures in six different concentrations and kept at 37$^{o}$C and 5% $CO_2$ for three days. After that, viability of cells in cultures was examined by calcein assay. The concentration killing 50% of tumour cells will be calculated.

**RESULTS.** Rigid and flexible docking techniques were used for binding modes analysis and design of new inhibitors. X-ray structures of three (ATP, olomoucine, roscovitine) cdk2 complexes were available at the beginning of the study and were used to optimize docking parameters. Then the new potential inhibitors were docked into the cdk2 enzyme, the enzyme/inhibitor interaction energies were calculated and tested against cdk1 and cdk2 assayed activities. A significant rank correlation between the activity and the rigid docking interaction energy has been found. This data led to further development of potent inhibitors like olomoucine II, with strong cdk1 and cdk2 inhibitory activity and strong cytotoxicity for different tumor cell lines (Tab. 1).

| Compound | $IC_{50}$ (μM) | | $GI_{50}$ (μM) | | | |
|---|---|---|---|---|---|---|
| | cdk1 | cdk2 | MCF7 | K562 | 3T3 | CEM |
| 6-(3-hydroxybenzylamino)-2-[[(1-hydroxymethyl)-2-(methyl)propyl]amino]-9-isopropylpurine | 0.08 | 0.03 | 16.8 | 11.3 | 17.1 | 5.5 |
| 6-(2-hydroxybenzylamino)-2-[[(1-hydroxymethyl)-2-(methyl)propyl]amino]-9-isopropylpurine | 0.05 | 0.009 | 7.7 | 27.5 | 11.2 | 9.6 |
| 6-(3-hydroxybenzylamino)-2(R)-[[(1-hydroxymethyl)propyl]amino]-9- isopropylpurine | 0.1 | - | 33.6 | 27.2 | 22.2 | 11.5 |
| 6-benzylamino-2-[[(1-hydroxymethyl)-2-(methyl)propyl]amino]-9-isopropylpurine | 0.8 | - | 135 | > 167 | > 167 | > 167 |
| 6-benzylamino-2-(2-hydroxyethylamino)-9-methylpurine | 7 | 1.2 | 129 | > 167 | 124 | 46.8 |
| 6-benzylamino-2(R)-[[(1-hydroxymethyl)propyl]amino]-9- isopropylpurine | 0.65 | 0.38 | 14 | 51.5 | 15 | 5.23 |

**Table 1.** Comparison of $IC_{50}$ and $GI_{50}$ Values for Various Purine Derivatives Tested on cdk1, cdk2 and several Tumour Cell Lines.

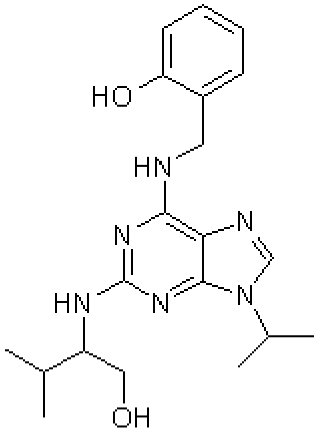

**Fig. 1.** Chemical structure of olomoucine II (6-(2-hydroxybenzylamino)-2[[1-(hydroxymethyl)-2-(methyl)propyl]amino]-9-isopropylpurine).

**DISCUSSION.** It implies that (i) the rigid docking can be used as a tool for qualitative prediction of activity and (ii) values obtained by the rigid docking technique into cdk2 active site can also be used for the prediction of cdk1 activity. While the resulting geometries obtained by the rigid docking are in a good agreement with the X-ray data, the flexible docking not always produced the same inhibitor conformation as found in the crystal.

**ACKNOWLEDGEMENT.** This work is supported Czech Ministry of Education via grant MSM 153100008.

**REFERENCE.**

1. Havlíček, L., Hanuš, J., Veselý, J., Leclerc, S., Meijer, L., Shaw, G., and Strnad, M. (1997) J. Med. Chem. 40, 408-412
